# Ethical and Clinical Boundaries in Genomics & Newborn Screening: A Brief Report from IPIC2025

**DOI:** 10.3390/ijns12020043

**Published:** 2026-06-18

**Authors:** Raquel Yahyaoui, Lúcia Mamede, Martin Zach, James Taylor, Claire Booth, Rosalind Fisher, Věra Franková, Adli Ali, Johan Prevot, Martine Pergent, Roberta Anido de Pena, Elizabeth Rivers

**Affiliations:** 1 Laboratory of Metabolic Disorders and Newborn Screening Center of Eastern Andalusia, Hospital Regional Universitario de Málaga, 29011 Malaga, Spain; 2 Biomedical Research Institute of Málaga, IBIMA-Plataforma BIONAND, 29590 Malaga, Spain; 3 Departamento de Biomedicina y Odontología, Facultad de Ciencias Biomédicas y Deporte, Universidad Europea de Andalucía, 29010 Malaga, Spain; 4 International Patient Organisation for Primary Immunodeficiencies (IPOPI), 1050 Brussels, Belgium; lucia@ipopi.org (L.M.);; 5 Department of Analytic Philosophy, Institute of Philosophy of the Czech Academy of Sciences, Jilská 1, 110 00 Prague, Czech Republic; 6 School of Humanities and Social Sciences, The College of New Jersey, Ewing, NJ 08628, USA; 7 Department of Paediatric Immunology and Gene Therapy, Great Ormond Street Hospital for Children, NHS Foundation Trust, London WC1N 3JH, UK; 8 Great Ormond Street Institute of Child Health, University College London (UCL), London WC1N 3JH, UK; 9 Department of Clinical Immunology, Oxford University Hospitals NHSFT, Oxford OX3 9DU, UK; 10 Institute of Medical Biochemistry and Laboratory Diagnostics, First Faculty of Medicine, Charles University, 121 08 Prague 2, Czech Republic; 11 Department of Pediatric, Faculty of Medicine, Universiti Kebangsaan Malaysia, Kuala Lumpur 56000, Malaysia; 12 Research Center, Hospital Tunku Ampuan Besar Tuanku Aishah Rohani, Universiti Kebangsaan Malaysia (UKM) Specialist Children’s Hospital, Kuala Lumpur 56000, Malaysia

**Keywords:** newborn screening (NBS), population screening, ataxia–telangiectasia, severe combined immunodeficiency (SCID), genomics, incidental detection, ethics

## Abstract

The Ethics Session of the International Primary Immunodeficiency Congress (IPIC), held in November 2025 and organised by the International Patient Organisation for Primary Immunodeficiencies (IPOPI), examined one of the most challenging developments emerging from genomic-enhanced newborn screening: the identification of serious, non-treatable disorders as incidental detection in programmes originally designed to detect severe combined immunodeficiency (SCID). Using a fictionalised clinical scenario based on the recent literature, the session explored the early diagnosis of ataxia–telangiectasia (AT) detected through TREC-based screening. The discussion highlighted the clinical value and psychological risks associated with presymptomatic detection, the persistent shortcomings in parental consent processes, the systemic pressures created by expanding genomic testing, and the ethical challenges surrounding the reporting and management of incidental findings in screening. The debate underscored the need for internationally coordinated frameworks to guide the management of incidental detection in newborn screening as genomic technologies become more deeply embedded in routine public health practice.

The latest International Primary Immunodeficiency Congress (IPIC), hosted by the International Patient Organisation for Primary Immunodeficiencies (IPOPI), was held in November 2025 in Prague, Czech Republic. This clinical congress, attended by a global audience, highlighted current advances in the field of primary immunodeficiencies (PID) and associated conditions. As part of the programme, the session “Ethics Session: Philosophical and Ethical Considerations to Clinical Boundaries in Genomics,” centred on a question that increasingly defines the evolution of newborn screening (NBS) in a genomic era: how should healthcare systems handle the off-target findings of severe, non-curable diseases in population-based newborn screening programmes designed to identify other conditions? A fictional case, closely aligned with the scientific literature on early identification of ataxia–telangiectasia (AT) through NBS-SCID (Severe Combined Immunodeficiency) [1], provided the basis for a broad reflection on the clinical, ethical, and societal dimensions of incidental detection in this programme. The session involved a multidisciplinary group including experts in philosophy and bioethics, paediatric immunology, clinical immunology, laboratory diagnostics, and genomic medicine from academic institutions and hospitals in the Czech Republic, the United Kingdom, the United States and Malaysia. The session was attended by leading scientific and medical experts, patient organisation representatives, nurses, industry representatives, and other key stakeholders, including representatives of the International Society for Neonatal Screening (ISNS).

Importantly, current SCID newborn screening programmes are generally based on quantification of T-cell receptor excision circles (TRECs), a functional molecular assay assessing T-cell production rather than genomic sequencing itself. However, the discussion also reflected broader debates regarding the increasing incorporation of genomic technologies into population-based newborn screening. The case involved a newborn girl screened at one week of life. Her TREC level fell within the non-urgent positive range, prompting confirmatory testing that revealed low T-cell counts, a hyper-IgM phenotype, and ultimately a biallelic pathogenic variant in the ataxia–telangiectasia mutated (ATM) gene. This pattern mirrors what has been described in several studies: many infants with AT already exhibit significantly reduced TREC values, and retrospective analyses suggest that approximately three-quarters of dried blood spots from patients with AT would fall below standard screening thresholds [1]. Although AT is not a primary target of SCID screening, its incidental detection is becoming more frequent as programmes expand internationally. A central ethical tension discussed was that identifying infants with SCID through TREC-based screening may inevitably also identify some infants with AT. This reflects a broader population-screening dilemma in which unintended harms or burdens may be accepted in order to achieve substantial benefit for another group.

A major point of discussion concerned whether conditions without effective treatment should be disclosed when they are incidentally detected during population screening. Although the classical Wilson and Jungner principles have traditionally emphasised the availability of an accepted treatment as a key criterion for screening, their interpretation has evolved and varies across jurisdictions. While some screening frameworks prioritise access to effective disease-modifying interventions, others also recognise clinical surveillance, avoidance of diagnostic delay, informed family decision-making, reproductive planning, and broader family benefit as meaningful outcomes. Subsequent reinterpretations in the genomic era have reflected this broader perspective [2]. The panel also acknowledged that these principles are increasingly challenged by the realities of genomic testing. Early detection of AT could prevent a prolonged diagnostic odyssey, help avoid exposure to ionising radiation, guide vaccination choices, identify carrier parents at increased cancer risk, and allow earlier recognition of infants with more severe immunological phenotypes [2,3,4]. From this perspective, even without a cure, early knowledge can enable clinical vigilance, anticipatory guidance, and family-level decision-making.

Nevertheless, the potential benefits coexist with legitimate concerns. Presymptomatic labelling in an otherwise healthy infant may generate significant psychological distress and alter the family’s early bonding experience. Moreover, the absence of proven disease-modifying interventions—and the uncertainty surrounding options such as early haematopoietic stem cell transplantation—highlights the fragility of any claim that early diagnosis is inherently beneficial. Existing data on transplantation in AT indicate substantial toxicity and mixed outcomes [3], raising the question of whether early detection risks burdening families with complex decisions without offering real improvement in prognosis.

The issues of information provision regarding the tests included in the NBS, the pathologies and potential outcomes, and consent were all raised during the ethical discussion. Despite the increasing complexity of screening, parental consent processes remain insufficiently robust. Empirical studies show that a notable proportion of mothers do not recall receiving meaningful information about newborn screening within months of giving birth [5]. This gap is even more problematic in the context of emerging genomic technologies, where future screening approaches may introduce additional complexity in result interpretation and communication. Participants also emphasised that newborn screening differs fundamentally from clinical diagnostic testing, as it operates as a population-based public health programme designed to maximise benefit while minimising harm at the population level. Participants also emphasised that consent in newborn screening differs fundamentally from consent for individual clinical diagnostic testing, as parents are consenting to participation in a population-based public health programme rather than to a personalised clinical investigation. The discussion suggested that consent should not be treated as a single event but rather as a staged process beginning during pregnancy and re-emphasised at the moment of sample collection and again during confirmatory testing. Such an approach would better align with the ethical requirement for genuine understanding and respect for parental autonomy while strengthening the trust with healthcare professionals. Attention to cultural differences in perceptions of genetic information and healthcare decision-making is also essential to ensure that consent processes are inclusive and respectful.

Beyond clinical and individual considerations, the session also addressed broader systemic and structural questions. Expanding NBS with genomic screening could considerably increase the number of diagnoses and generate a surge in demand for specialist services. In many countries, existing systems already struggle with workforce shortages, limited laboratory capacity, and insufficient funding [6,7,8]. There is a risk that an unplanned expansion of screening assays could threaten the sustainability of the entire programme. At the same time, participants recognised the ethical tension between limiting reporting because of system constraints and ensuring equitable access to potentially relevant health information. Early diagnosis may prevent costly downstream complications and can strengthen the case for investing in more robust immunology and genetic services. The tension between system capacity and patient rights emerged as one of the defining ethical challenges for the next decade of newborn screening. The discussion also highlighted that implementation decisions inevitably depend on local infrastructure, workforce capacity, and public health priorities, potentially contributing to differences between jurisdictions.

Finally, the idea of replacing TREC-based screening with first-tier whole-genome sequencing was considered. Despite technological progress, the consensus was that such a shift would currently be premature. The high incidence of variants of uncertain significance in SCID-associated genes may limit the sensitivity and clinical interpretability of a genomic-first screening approach compared with current functional screening strategies [5,8]. In addition, for several genes, including ATM, the lack of validated functional assays may hinder orthogonal confirmation even when pathogenic or likely pathogenic variants are identified. Functional screening methods, such as TREC quantification, therefore continue to play an indispensable role by identifying clinically meaningful T-cell lymphopenia regardless of genotype. Similar concerns have been raised in the literature on genomic newborn screening and public health ethics, particularly regarding interpretation, data governance, equity, and the potential impact on public trust in screening programmes [9].

Overall, the session highlighted how genomic technologies are forcing a reconsideration of long-standing assumptions about newborn screening. In the case of AT, early diagnosis sits at the intersection of potential clinical benefit, psychological burden, and unresolved ethical questions. As SCID screening continues to expand globally, the number of infants diagnosed presymptomatically with AT is expected to rise [1], making it imperative to develop shared guidance on communication, follow-up, and family support. The reflections offered at IPIC 2025 underscore the importance of sustained dialogue between clinicians, patient organisation representatives, screening experts, ethicists, policymakers, and families to ensure that genomic innovations translate into ethically sound and equitable screening practices.

## Data Availability

The original contributions presented in this study are included in the article. Further inquiries can be directed to the corresponding author.
